# Post-COVID-19 Barriers to Diabetic Retinopathy Screening Attendance: An Updated Systematic Review

**DOI:** 10.7759/cureus.96550

**Published:** 2025-11-11

**Authors:** Delwar Hussain, Mohd Javaid Iqbal

**Affiliations:** 1 Orthopaedics, Royal Blackburn Teaching Hospital, Blackburn, GBR; 2 Geriatrics, Salford Royal NHS Foundation Trust, Manchester, GBR

**Keywords:** barriers, diabetes mellitus, diabetic retinopathy, non-attendance, screening

## Abstract

Diabetic retinopathy (DR) is a preventable cause of vision loss; with screening, there is the capability to recognise and treat the condition early. However, screening compliance remains sub-optimal, and the COVID-19 pandemic caused widespread disruptions to the screening programme. This review aims to update prior systematic reviews to identify barriers that remain, as well as identify new barriers that may have occurred due to the pandemic. Following the Preferred Reporting Items for Systematic reviews and Meta-Analyses (PRISMA) 2020 guidelines, we searched seven databases (January 2020-July 2025) for English language primary studies on DR screening non-attendance, yielding 16 relevant studies across diverse regions. Key barriers fell into patient-related, health system, and environmental categories. Although there was evidence to suggest the same barriers remained, there is evidence to suggest the pandemic exacerbated prior barriers and introduced new barriers. These findings suggest the need for context-specific interventions to improve DR screening in the post-pandemic era.

## Introduction and background

Diabetic retinopathy (DR) is the most common microvascular complication of diabetes mellitus (DM) and continues to be one of the most significant causes of preventable vision loss [1,2]. Both the prevalence and burden of diabetes are expected to increase significantly over the next few decades, so it can be expected for a greater pressure on healthcare services to manage DR and its associated complications. Individuals with type 1 diabetes (T1DM), compared to patients with type 2 diabetes (T2DM), are at greater risk of developing DR [3,4].

The pathophysiology behind DR is driven by hyperglycaemia, leading to a cascade of events that includes retinal blood vessel dilatation, both pericyte and endothelial cell apoptosis, and microaneurysm formation [5]. DR is broadly categorised into non-proliferative diabetic retinopathy (NPDR), in which there are exudates, microaneurysms, and haemorrhages, but no new blood vessel growth and proliferative (PDR), which is associated with neovascularisation [6]. In regard to severity, DR can be further classified into five stages according to the International Clinical Disease Severity Scale: no apparent retinopathy, mild NPDR, moderate NPDR, severe NPDR, and PDR [6,7].

Systematic DR screening is essential to identify and treat the condition early, which can prevent up to 98% of severe vision loss [8]. For example, the NHS England Diabetic Eye Screening Programme (DESP) has illustrated its effectiveness by reducing DR certifications for WHO severe visual impairment from 5.5% to 3.5% across a 10-year period [9]. However, the COVID-19 pandemic had gross implications for diabetic screening worldwide. Both the effect of lockdowns and restrictions on established screening programmes lead to widespread delay in both community and hospital settings [10]. These disruptions raised concerns of delayed detection and increasing prospects of patients presenting more acutely with worse visual outcomes [11]. Early reports indicate lapses in eye care during the COVID-19 pandemic resulted in worse outcomes. For example, Zhou et al. (2022) suggested that patients who had experienced pandemic-related delays in appointments had fourfold higher odds of vision loss on follow-up compared to those seen on schedule [12]. Therefore, this review intends to update the barriers described in a prior systematic review [1]. This updated review will examine new primary research published since the beginning of 2020, with a focus on the new challenges brought on by the COVID-19 pandemic and the possible impact it may have had on DR screening globally. 

## Review

Methods

Eligibility Criteria

This systematic review was conducted and reported in accordance with the Preferred Reporting Items for Systematic reviews and Meta-Analyses (PRISMA) 2020 guidelines. Only publications that were available in English and published after 2020 were considered, ensuring they were both peer-reviewed and the full texts were available. Research that was included focused on both healthcare professionals’ and patients’ perspectives on non-attendance or barriers to diabetic retinopathy screening, to capture a wide array of possible causes of non-adherence. Studies were excluded if they did not directly address DR screening or attendance (e.g., studies that focused on other diabetes-associated complications) or if they were not primary research (such as audits). Both non-English publications and publications before 2020 were excluded. Furthermore, publications that focused on specific interventions to increase uptake of diabetic screening or purely economic evaluations of non-attendance were excluded. 

Search Strategy

A comprehensive literature search was completed across seven electronic databases to identify relevant studies. The databases searched were Medline, CINAHL Ultimate, Embase, Emcare, PubMed, the Cochrane Library, and Cochrane CENTRAL. The search spanned publications from January 1, 2020, up to July 2025. The search strategy was based on key concepts based on the systematic review and used a combination of these terms: "patients’ non-attendance", "ophthalmology/eye care", "screening/scheduling", "diabetes", and "healthcare access/utilisation". Relevant synonyms and related terms are applied for each search. The detailed terms used for each concept are presented in Table 1.

**Table 1:** Search terms

Index term	Synonyms	Related terms
Patients’ non-attendance	(1a) missed appointment; (1b) failed appointment; (1c) absent from clinic; (1d) no-show / no show; (1e) non-attendance	(1f) non-compliance; (1g) non-adherence; (1h) uptake; (1i) patient participation; (1j) refusal to participate
Ophthalmology / eye care	(2a) vision care; (2b) eye health; (2c) ocular care; (2d) retinal care	(2e) ophthalmic services; (2f) retina / retinal monitoring; (2g) visual screening
Screening / scheduling	(3a) screening programme; (3b) screening program; (3c) screening schedule	(3d) appointment system; (3e) follow-up schedule; (3f) recall system
Diabetes	(4a) diabetes mellitus; (4b) diabetic patient; (4c) T1DM; (4d) T2DM	(4e) IDDM; (4f) NIDDM; (4g) diabetic retinopathy; (4h) diabetic maculopathy
Healthcare access / utilisation	(5a) Health services accessibility; (5b) appointment and schedules; (5c) no-show patients; (5d) patient acceptance of healthcare	(5e) barriers to access; (5f) patient engagement; (5g) patient attendance; (5h) treatment refusal

The terms that were used were adjusted for each database’s indexing system (e.g., using MeSH terms in Medline and Emtree in Embase) and combined using Boolean operators (AND/OR) to ensure a coherent search. For example, synonyms used for patient non-attendance were "missed appointment", "no-show", "nonattendance", or "non-compliance", combined with terms for the eye screening context (e.g., "diabetic retinopathy screening", "eye exam", "vision care", or "screening programme", along with terms for diabetes (e.g., "diabetes mellitus", "T1DM"/"T2DM") and healthcare access factors (e.g., "health services accessibility", "barriers to access", "patient attendance").

Study Selection

All retrieved records were imported into Rayyan, a web-based application designed to assist with systematic review screening [13]. Initially, 138 duplicates were identified and removed at the start of this process. Two independent reviewers screened the remaining abstracts to identify articles that fit the inclusion criteria and were relevant to the review. Both reviewers were blinded to each other’s decisions. Conflicts were resolved through discussions to ensure a consensus was achieved. 

Inclusion criteria were as follows: studies on patients with type 1 or 2 diabetes, studies published from 2020 onwards, belonging to a dedicated diabetic eye screening programme, and examining reasons/perspectives for patient non-attendance to screening appointments. Exclusion criteria were as follows: studies published before 2020, foreign language studies, audits and quality improvement projects (QIPs), interventional studies that seek to improve uptake of attendance to screening programmes or focus solely on patient education, and examining non-attendance to appointments outside of diabetic eye screening programme (i.e. ophthalmology clinic appointments).

**Figure 1:**
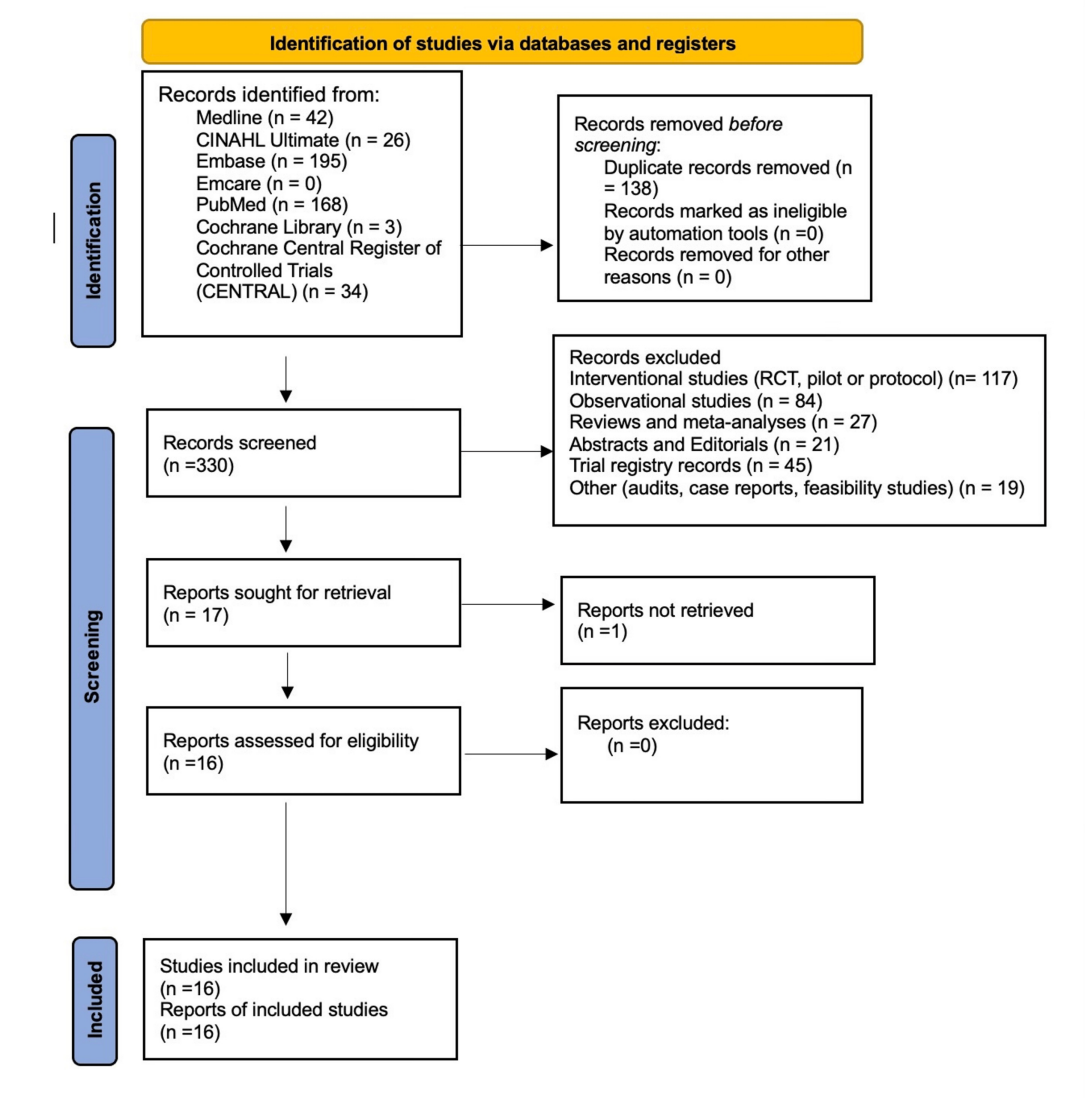
Preferred Reporting Items for Systematic reviews and Meta-Analyses (PRISMA) 2020 flow diagram of the study identification and selection.

Following this, the full-text papers were independently analysed by the same two reviewers, each analysed against the eligibility criteria. Full texts were evaluated against the screening criteria, with a specific reason for exclusion. Again, any conflicts at the full-text screening stage were again settled by discussion among the two reviewers. To ensure that each paper was appraised appropriately and to reduce the risk of bias, the Critical Appraisal Skills Programme (CASP) checklists were utilised (Tables 2, 3, 4)[14]. Studies were grouped by methodology (qualitative, quantitative, mixed methods). Each CASP criterion is scored 0 (not met), 1 (partially met), or 2 (fully met). Total scores were assessed (out of 20) to reflect overall quality; all studies scored between 16 and 20, indicating generally high quality. Sixteen papers were identified and selected for further data extraction. The overall process is summarised in the following PRISMA flowchart and CASP checklist summaries for each study (Figure 1).

**Table 2:** Qualitative studies: CASP checklist summary Critical Appraisal Skills Programme (CASP) qualitative study criterion [14]: 1. Was there a clear statement of the aims of the research? 2. Is a qualitative methodology appropriate? 3. Was the research design appropriate to address the aims of the research? 4. Was the recruitment strategy appropriate to the aims of the research? 5. Was the data collected in a way that addressed the research issue? 6. Has the relationship between the researcher and participants been adequately considered? 7. Have ethical issues been taken into consideration? 8. Was the data analysis sufficiently rigorous? 9. Is there a clear statement of findings? 10. How valuable is the research?

Study (Author, Year)	Q1	Q2	Q3	Q4	Q5	Q6	Q7	Q8	Q9	Q10	Total
Chauhan et al. 2025 [15]	2	1	1	2	2	1	1	2	2	2	17
Kumar et al. 2020 [16]	2	2	2	2	2	1	2	1	2	2	18
Prothero et al. 2022 [17]	2	2	2	2	2	2	1	2	2	2	19
van Allen et al. 2020 [18]	2	2	2	2	2	1	2	1	2	2	18
Yahya et al. 2020 [19]	2	2	2	2	2	2	1	2	2	2	19

**Table 3:** Quantitative studies: CASP checklist summary critical appraisal skills programme (CASP) quantitative study criterion [14]: 1. Did the study address a clearly focused issue (research question)? 2. Was the study design appropriate to answer this question? 3. Were the participants (cohort/sample) recruited in an acceptable way? 4. Were the key exposures/factors measured accurately (to minimise bias)? 5. Was the outcome (screening adherence) measured accurately (to minimise bias)? 6. Did the study identify all important confounding factors? 7. Did the analysis take adequate account of confounding factors? 8. Are the results clearly presented and meaningful? 9. How precise are the results (e.g., were confidence intervals or p-values provided)? 10. Can the results be applied to the broader population (external validity)?

Study (Author, Year)	Q1	Q2	Q3	Q4	Q5	Q6	Q7	Q8	Q9	Q10	Total
Anvari et al. 2024 [20]	2	2	2	2	2	1	1	2	1	2	18
Bruggeman et al. 2021 [21]	2	2	1	2	1	1	1	2	1	2	17
Kelly et al. 2021 [22]	2	2	2	2	2	2	2	2	2	2	20
Kuo et al. 2020 [23]	2	2	2	2	2	1	2	2	1	2	18
Lawrenson et al. 2020 [24]	2	2	2	2	2	2	1	2	1	2	19
O’Keeffe et al. 2021 [25]	2	2	2	2	2	1	2	2	1	2	18
Olvera-Barrios et al. 2021 [26]	2	2	2	2	2	2	1	2	1	1	19
Owusu-Afriyie et al. 2023 [27]	2	2	1	1	1	1	0	2	1	1	16
Yusuf et al. 2020 [28]	2	2	2	2	2	2	1	2	1	1	19

**Table 4:** Mixed methods studies: CASP checklist The authors devised an adapted Critical Appraisal Skills Programme (CASP) checklist to be used for appraisal of mixed-methods studies to appropriately evaluate both their qualitative and quantitative components, ensuring balanced appraisal of findings. Adapted CASP Criteria: 1. Was there a clear statement of the research aims? 2. Was a mixed methods approach appropriate for addressing the aims? 3. Was the quantitative component of the study appropriately designed and conducted? 4. Was the qualitative component of the study appropriately designed and conducted? 5. Was the sampling strategy suitable for both the quantitative and qualitative parts? 6. Were data collection methods appropriate to address the research questions (for both components)? 7. Was the data analysis rigorous for both quantitative and qualitative data? 8. Were the quantitative and qualitative findings integrated effectively? 9. Is there a clear statement of the overall findings (incorporating both components)? 10. Did the mixed‐methods study provide valuable insights (did using both methods add useful knowledge)?

Study (Author, Year)	Q1	Q2	Q3	Q4	Q5	Q6	Q7	Q8	Q9	Q10	Total
Cleland et al. 2022 [29]	2	2	2	2	2	2	2	1	2	2	19
Curran et al. 2024 [30]	2	2	2	2	2	2	1	1	2	2	18

Data Extraction 

Relevant data were extracted and imported into an MS Excel spreadsheet (Microsoft Corp., USA) for each included study. This information included bibliographic information (author, year of publication), the country and setting of the study, the population's characteristics (e.g. sample size, age range, type of diabetes), the study's design and methodology, and the main outcomes/findings related to diabetic eye screening attendance or non-attendance. Both barriers and predictors to DR screening that were identified were also incorporated into the spreadsheet to facilitate both the results and discussion.

Results

Several barriers to diabetic retinopathy screening adherence were identified. These can broadly be categorised into the following: patient-related factors (age, gender, socioeconomic status, knowledge, cultural attitudes), health system barriers (service provision and clinic capacity), and environmental factors (social support and pandemic disruptions). A summary of the key points has been collated into Table 5: 

**Table 5:** Summary of the included studies' characteristics

Studies	Demographics	Population size	Methods used	Aim	Key points
Lawrenson et al. 2020 [24]	Adults with T1/T2DM in 3 UK NHS programmes	97,048	Retrospective cohort	Examine attendance trends in DR screening	80% screened <12 months. 88% by 36 months. Non-attendance higher in young, deprived, ethnic minorities.
O’Keeffe et al. 2021 [25]	Adults with T2DM in 30 Irish GP practices over age of 18	1,106	Retrospective observational	Estimate uptake of DR screening and Identify factors associated with attendance.	78% attended in 12 months. Predictors of attendance include district nurse review in the last 12 months and on target blood glucose control. Non-attendance was linked to female sex.
Kelly et al. 2021 [22]	National DR programme participants, Ireland	158,655	Retrospective cohort	Determine patient and screening level factors associated with screening non-attendance.	52% of non-attenders were from those who missed 3 or more appointments. T2DM patients had higher rates of non-attendance. Weather may influence attendance.
Olvera-Barrios et al. 2021 [26]	Multi-ethnic London diabetic population aged >12 years old	84,449	Retrospective cohort	Examine the association of sociodemographic characteristics with attendance at diabetic eye screening.	Small differences in screening attendance rates between ethnicities. Asian ethnicity associated with higher rates of attendance compared to White British. Poorer socioeconomic profile associated with non-attendance.
Prothero et al. 2022 [17]	UK young adults (18–34) with T1DM	29	Semistructured Qualitative interviews	Identify barriers and enablers of diabetic eye screening.	Key barriers included lack of understanding of the importance of DR screening, lack of support following DR screening results and resource related barriers such as appointment scheduling inflexibility.
Bruggeman et al. 2021 [21]	USA youth (10–26 years old) with T1DM diagnosis of ≥5 yrs	271	Cross-sectional study (patient reported questionnaire)	Identify barriers to DR screening in youth and young adults with T1DM.	Missed school/work was the most common barrier reported (20.7%). 62.1% of respondents adhered to recommended screening guidelines.
van Allen et al. 2020 [18]	Immigrants with T2DM (Pakistan, China, Africa/Caribbean)	39	Qualitative study (interviews)	Identify barriers/enablers to eye screening in groups of immigrants to Canada.	Barriers include fear of harm from screening, cost of screening, low awareness, language barriers, preference to one’s own country of birth for screening, winter weather and preference for alternative medicine.
Owusu-Afriyie et al. 2023 [27]	Papua New Guinea diabetic patients booked for eye clinic at MPHEC	104	Cross Sectional Study (Questionnaire)	Determine the barriers to the uptake of DR screening in Papua New Guinea	Main barriers to screening included time constraints, poor awareness of DR and long waiting times at DR screening centres. 63.5% of respondents had discontinued using diabetic pharmacotherapy.
Cleland et al. 2022 [29]	Patients registered under Kilimanjaro diabetic eye screening programme.	300 in quantitative study + 33 in qualitative study	Mixed-methods	Explore gender biases amongst screening programme patients	Quantitative analysis demonstrated lower levels of education and tertiary hospital appointment attendance amongst female participants. Qualitative analysis showed good understanding of threat to vision from DR but poor understanding of disease chronicity.
Yahya et al. 2020 [20]	Palestinian adults non-compliant with DR screening.	24	Qualitative Study (Focus groups)	Explore patient reported barriers to DR screening	Reports financial barriers such as cost of examination, treatment and transportation. System barriers include lack of resources. Personal barriers include fear of results and anecdotal negative experiences. Cultural barriers include the stigma of wearing glasses and asymptomatic testing.
Yusuf et al. 2020 [28]	US adults referred for first DR screening in Yale-New Haven medical register	1,397	Retrospective cohort	Determine if neighbourhood deprivation is associated with adherence to ophthalmic DR evaluation.	52% attended dilated eye examinations. Higher rates of rate deprivation were associated with non-adherence.
Kuo et al. 2020 [23]	Adults with T1 or T2DM at Barnes Jewish Hospital.	974	Retrospective Cohort	Identify rate of adherence to eye screening. Identify factors associated with adherence.	Only 33.9% patients were adherent to eye screening in a two year period. Improved screening attendance was associated with female sex, older age, primary language other than English and attendance at other diabetic clinics.
Kumar et al. 2020 [16]	Indian adults (aged >50) with T2DM (>5yrs) and no diagnosis of DR.	15 patient interviews and 8 health care professional interviews.	Qualitative Study (interviews)	Explore understanding of and barriers to diabetic retinopathy screening from the perspectives of patients and healthcare providers.	“Four themes that best explained the data were recognising and living with diabetes, care-seeking practices, awareness about diabetic retinopathy and barriers to diabetic retinopathy screening.” [28]
Chauhan et al. 2025 [15]	Diabetes patients and health care professionals (HCPs) in North India	26 patients + 19 HCPs	Qualitative/Cross sectional Study (interviews)	Explore perspective of patients and HCPs on barriers to diabetic eye screening.	Reports financial barriers due to insurance unawareness and constraints of employment. Structural barriers due to lack of resources and access to DR screening. Educational and psychological barriers such as misconceptions of screening and mistrust of health system.
Anvari et al. 2024 [20]	T2DM patients reporting for four tertiary hospital non-ophthalmology clinics in Iran.	240	Cross-sectional Study (Questionnaire)	Evaluate the adherence rate to DR screening program. Identify the characteristics associated With non-compliance.	Non-compliance rates higher than pre-pandemic figures. Socioeconomic factors such as transportation may affect adherence. Healthcare promotion of eye exams increased adherence. “The COVID-19 pandemic emerged as a prominent barrier, with numerous patients citing it as a reason for their non-compliance due to disruptions in clinical care.” [25]
Curran et al. 2024 [30]	Diabetic patients (12-26 years old) registered at Women and Children hospital in Dhaka.	390	Mixed-methods	Identify the factors affecting attendance or non-adherence to DR screening among children and young adults with diabetes mellitus in Bangladesh.	Reports high rates of attendance (88%). Adherence to screening was highest amongst younger patients (<15years old), females and those with higher HbA1c levels. Barriers include cost, service accessibility and transportation.

Patient-Related Factors

Younger age remained a key theme, as a factor that was associated with poor attendance at screening. In the UK, it was reported that patients younger than 35 years of age often had poor compliance with screening. Furthermore, 20% of 18-34 year olds were unscreened at three years [24]. An age-related theme was also found in the Irish national screening programme, as the risk of non-attendance increased by 24% for each decade further from 70 [22]. A gender discrepancy was also observed, although it varied by location. In Tanzania, it was witnessedthat women were less likely to attend DR screening; this was partially attributed to traditional gender roles in which men often controlled finances and healthcare-related decisions [29]. However, it was observed in Bangladesh that men were less likely to attend (odds ratio 0.29 between sexes) [30]. Furthermore, there appeared to be a language barrier component to low attendance compliance. As communities in Canada demonstrated both language barriers and a patient preference for eye care in their home country versus Canadian services [18]. Socioeconomic factors had both implications in high-income countries and low-income countries. In the United States (US), patients in neighbourhoods that were considered deprived were much less likely to attend their first screening appointment [28]. Similarly, in London, greater area deprivation was associated with poor adherence to the screening programme [26]. Financial hurdles were also evident in Palestine, in which evidence suggested that lack of insurance and transportation costs led to poor compliance with screening [19]. Knowledge and patient perception appeared to be a key theme that was observed from the data. Patients did not understand the importance of attending screening whilst being asymptomatic [17]. Misconceptions and fear also deterred patients: some feared screening or potential laser treatment, or had anxiety from seeing family members go blind despite care [19]. An element of anxiety appeared to be a reported cause of poor adherence, as patients admitted to having feelings of being overwhelmed [15].

Health System Barriers

Healthcare shortcomings were reported to be limiting screening uptake. In both Palestine and Papua New Guinea clinics, where screening programmes have limited capacity and long waiting times, these discouraged patient adherence [19,27]. This was also a common theme, which was evident in North Indi,a as there was inadequate infrastructure and trained professionals to deliver the screening [15]. Scheduling and logistic factors appeared to be an issue in both the UK and US, as patients described difficulty attending because of rigid appointment times, clashes with work or school, and challenges rescheduling missed appointments [17,21]. Another important barrier which was raised was the failure of physicians to recommend screening. This was seen in Iran, where the lack of referrals for annual eye examinations was strongly linked with non-adherence [20].

Environmental Factors

A common recurrent theme that occurred was the lack of transport, as patients sometimes did not have their own mode of transport, needed family members for assistance, or there were no adequate local facilities [15,29]. There is also evidence that COVID-19 provided some disturbance to adequate compliance with the screening programme. Moreover, 38% of non-adherent patients in Iran attributed missed screening to fear of infection or service disruptions [20].

Discussion

Whilst comparing the pre-pandemic findings of Kashim et al. [1], it is evident that core barriers to diabetic screening adherence remain largely consistent, although new challenges are present. Patient-related factors such as young age, low socioeconomic status, poor disease awareness, or perception were determinants to poor compliance pre- and post-pandemic. Similarly, health system concerns remain. Infrastructure and poor service capacity continue to affect screening attendance as they did in earlier years. However, it is crucial to note the pandemic did exacerbate pre-existing factors that contributed to screening appointment attendance. For instance, patients who had already suffered due to socioeconomic issues in the pre-pandemic era continue to struggle, possibly due to the worsening economic situation in the pandemic [28]. Likewise, patient groups who were considered less likely to attend (younger patients, patients from ethnic minorities or socially marginalised groups) continued to struggle with compliance to diabetic eye screening. Reasons for this include communication issues, as those patients with language barriers may struggle to cope with changes in screening appointments due to the pandemic [24].

The pandemic introduced new issues, which contributed to reduced compliance with the screening programme. The fear of infection created a new psychological barrier to prevent attendance. COVID-19 in healthcare settings has deterred patients from attending [20]. Furthermore, the suspension of screening programmes during the pandemic also proved to be an issue, causing delayed or missed appointments [10]. This underlines the importance of health care systems needing plans in place for chronic disease screening during public health crises. It is important to note that the prior study in 2018 was mostly dominated by studies performed in Western countries [1]. This limited its application to countries which had larger financial resources and established screening programmes. Meanwhile, this review drew data from both low- and middle-income regions in Africa and Asia. This highlighted global barriers (e.g., socioeconomic hardship, young age) and context-specific ones (e.g., patriarchal norms, weak infrastructure). Regional differences in how the pandemic affected screening programmes highlight the importance that barriers must be contextualised to local healthcare systems.

Limitations

This review was restricted to the English language only; this language selection may potentially bias results as it could potentially exclude relevant data from non-English speaking countries. Hence, vital insights from large diabetes populations in those regions may not be considered. This may limit the global applications of the conclusions. In some studies, there may be aspects of recall bias or social desirability bias, as those studies relied on interviews, which required self-reported reasons for not being able to attend [15-17,19-21,27,29,30]. Whilst other studies were at risk of sampling bias, as those studies were conducted in single centres or urban tertiary clinics [16,19,23,26-28,30]. This may skew the findings towards the circumstances of those sites. Finally, the timeframe of the review may be considered short in terms of post-pandemic recovery, as it may be too early to fully assess long-term behavioural changes. There is also a risk of publication bias; studies highlighting possible barriers associated with COVID-19 could amplify the perceived impact of the pandemic on poor compliance in the literature.

## Conclusions

This review aimed to examine barriers to diabetic eye screening attendance after the COVID-19 pandemic. This updated review demonstrates that well known barriers continue to remain and that the pandemic had a compound effect on the known barriers whilst adding new challenges. The data reiterates long standing obstacles whilst also highlighting the issues in low-income countries through additional evidence included in this review. In future practice, health care systems should implement strategies to address both pre- and post- pandemic barriers such as flexible scheduling, proactive outreach to at risk groups, and contingency plans to maintain screening during public health crises.
